# Spontaneous Uterine Perforation and the Diagnostic Value of Laparoscopy: A Case Report and Literature Review

**DOI:** 10.7759/cureus.83596

**Published:** 2025-05-06

**Authors:** Kennedy Hess, Sarang Kashyap

**Affiliations:** 1 Medical School, Lincoln Memorial University DeBusk College of Osteopathic Medicine, Harrogate, USA; 2 Surgery, Raleigh Advanced Surgery, Raleigh General Hospital, Beckley, USA

**Keywords:** clinical diagnostic value, diagnostic laparoscopy, pyometra rupture, spontaneous uterine rupture, surgical acute abdomen

## Abstract

Spontaneous uterine perforation due to pyometra, the accumulation of fluid within the uterus, is an uncommon cause of acute abdomen. Malignancy should be ruled out in these cases, as pyometra is often associated with gynecological cancers. Symptoms of perforated pyometra are often vague and mistaken for gastrointestinal perforation. A 74-year-old postmenopausal woman presented with severe abdominal pain and nausea. Due to the nonspecific symptoms and pneumoperitoneum on CT imaging, preoperative assessment was consistent with perforated bowel; however, upon diagnostic laparoscopy and peritoneal lavage, perforated pyometra was found. A total hysterectomy with bilateral salpingo-oophorectomy was subsequently performed. Though rare, perforated pyometra should be considered in postmenopausal females with symptoms of acute abdomen due to its high morbidity and mortality. Diagnostic laparoscopy is effective for diagnosing unclear etiologies of acute abdomen and has many benefits compared to exploratory laparotomy.

## Introduction

Uterine perforation is an uncommon cause of acute abdomen and is often associated with intrauterine device use or iatrogenic causes; however, perforation can be spontaneous due to pyometra, the accumulation of purulent fluid within the uterus [1]. Causes of pyometra include gynecological malignancies, endometrial polyps, benign tumors, infections, intrauterine devices, cervical stenosis, and other idiopathic causes [1, 2]. Common symptoms of pyometra include vaginal discharge, postmenopausal bleeding, and lower abdominal pain, though 50% of women without perforation are asymptomatic [3, 4]. Perforation of pyometra is caused by impaired drainage of the uterus and a subsequent buildup of fluid and pressure, leading to the fundus being the most common site of perforation [2, 5]. This can lead to peritonitis, causing abdominal tenderness, rigidity, and/or guarding [3]. Here, we discuss a case of spontaneous perforation of pyometra leading to peritonitis diagnosed via diagnostic laparoscopy.

## Case presentation

A 74-year-old post-menopausal woman with hypertension presented to the Emergency Department with severe abdominal pain associated with nausea, but no vomiting. She complained of lower abdominal pain for the last month and chronic constipation, for which she had been taking laxatives. Her last bowel movement was two to three days prior, and she was no longer passing flatus. She denied any fever but noted sweating. She had a previous gastric bypass surgery in the 1970s and a laparoscopic appendectomy several years ago. On exam, she appeared ill and diaphoretic. Vitals upon presentation were as follows: body temperature 97.6°F, heart rate 70 beats/minute, blood pressure 120/60 mmHg, respiratory rate 18 breaths/minute, and O2 saturation of 98%. Abdominal exam revealed tenderness in the lower abdomen with guarding but without rigidity or distension. CT scan showed free air under the diaphragm and thickened colon walls, leading to the preoperative diagnosis of peritonitis with possible hollow viscus perforation. White blood cell count, neutrophils, and lactic acid were all elevated, supporting infection (Table 1).

**Table 1:** Pre-operative lab values suggestive of infection.

	Value	Reference Range
WBC	16.9	4.5-11 thousand/mm^3^
RBC	3.13	Female: 3.5-5.5 million/mm^3^
Hemoglobin	9.6	Female: 12-16 g/dL
Hematocrit	28.1	Female: 36%-46%
Mean Corpuscular Volume	90	80-100 µm^3^
Mean Corpuscular Hemoglobin	31	25-35 pg/cell
Mean Corpuscular Hemoglobin Concentration	34	31%-36% Hb/cell
Red Cell Distribution Width	15.5	12%-15%
Platelet Count	404	150-400 x 10^9^/L
Neutrophil %	90.7	55%-70%
Lymphocytes %	7.3	25%-33%
Monocytes %	1.1	3%-7%
Eosinophils %	0.1	1%-3%
Basophils %	0.3	0%-0.75%
Segmented Neutrophils	91	54%-62%
Sodium	139	136-146 mEq/L
Potassium	3.5	3.5-5.0 mEq/L
Chloride	113	95-105 mEq/L
Carbon Dioxide	16	33-45 mm Hg
Anion Gap	14	4-12 mEq/L
Blood Urea Nitrogen	37	7-18 mg/dL
Creatinine	1.5	0.6-1.2 mg/dL
Estimated Glomerular Filtration Rate	36	> 90
Glucose	198	< 140 mg/dL
Lactic Acid	4.1	< 2 mmol/L
Calcium	9.5	8.4-10.2 mg/dL
Total Bilirubin	0.3	0.1-1.0 mg/dL
Aspartate Aminotransferase	12	12-38 U/L
Alanine Aminotransferase	17	10-40 U/L
Total Alkaline Phosphatase	117	25-100 U/L
Total Protein	5.6	6.0-7.8 g/dL
Albumin	2.1	3.5-5.5 g/dL
Globulin	3.5	2.3-3.5 g/dL
Lipase	23	13-60 U/L

**Figure 1:**
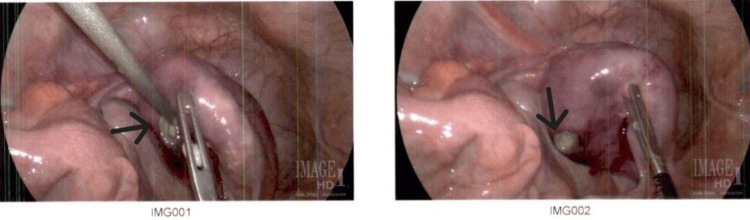
Intraoperative photos of fundal perforation with purulent fluid visible (black arrows).

We decided to perform a diagnostic laparoscopy to ascertain the origin of the perforation. 600 mL of purulent fluid was found within the abdomen and pelvis. Fluid was sent for culture, followed by intraperitoneal lavage. The stomach and small and large bowels were thoroughly inspected. No perforation was found, but the uterus was enlarged, erythematous, and edematous. Upon further examination, a 1.0 x 1.0 cm defect was found in the posterior uterine fundus (Figure 1). A total abdominal hysterectomy with bilateral salpingo-oophorectomy was subsequently performed. Histopathological studies revealed no evidence of malignancy, but there was purulent, necrotic material within the uterine cavity; cultures grew *Bacteroides fragilis*. The patient had a prolonged hospital stay complicated by *Escherichia coli* bacteremia and urinary tract infection, which were treated with broad-spectrum antibiotics.

## Discussion

The incidence of pyometra is higher in post-menopausal women, at 13.6%, while the incidence of spontaneous pyometra rupture is extremely rare, at 0.01- 0.05% [5, 6]. Biller et al have reported an increased incidence of rupture with comorbidities, such as diabetes, immunocompromised states, incontinence, obesity, and malnutrition [5]. Chan et al have reported that malignancy is associated with 35% of cases of ruptured pyometra, and a vast majority have been reported as idiopathic [7]. Our patient had no evidence of malignancy upon histopathological examination, which correlates well with findings of the other authors [7]. The most common bacterial agents cultured from pyometra are *Escherichia coli*, *Bacteroides fragilis*, and other anaerobes [5].

Perforated pyometra is often associated with a poor prognosis and reported mortality rates as high as 25% [8]. It is thus important to make a quick and accurate diagnosis so proper management, surgical or not, can be implemented. Unfortunately, diagnosing ruptured pyometra pre-operatively is a difficult task. Less than 10% of cases of pyometra present with the classic signs and symptoms [3]. Acute abdomen is the most common presentation, and imaging often shows pneumoperitoneum [3, 9]; therefore, it is often mistaken for gastrointestinal perforation. Only about 30% of cases are properly diagnosed preoperatively [2]. Diagnostic laparoscopy can be performed for an acute abdomen when the diagnosis is uncertain. Firat et al recommended diagnostic laparoscopy as it allows the surgeon to directly visualize and assess the injury [10]. Diagnostic laparoscopy can further aid in the completion of the therapeutic maneuvers, depending on the surgeon's expertise and patient factors. Diagnostic laparoscopy helps to determine the management course and reduce unnecessary laparotomies; thus reducing the time hospitalized, and overall recovery time [10]. In most cases, patients can be treated effectively laparoscopically, further decreasing the need for laparotomy [10].

Hysterectomy, as was performed in this patient, is the standard management for perforated pyometra [11]. It has been argued that without evidence of malignancy, surgeons should attempt to preserve the uterus. Browne has reported their method of hysteroscopy and uterine lavage to manage perforated pyometra and preserve the uterus when there was no evidence of malignancy [11]. On the other hand, Yousefi et al managed their case with uterine drainage, peritoneal lavage, and closure of the defect, followed by broad-spectrum antibiotics to preserve fertility [1]. As a joint intraoperative decision with gynecology and the surrogate decision maker, our postmenopausal patient received a total hysterectomy with bilateral salpingo-oophorectomy due to concern for malignancy.

## Conclusions

Although gastrointestinal perforation is the most common cause of perforation, peritonitis, pyometra, and uterine perforation should be included in the differential diagnosis for post-menopausal women with an acute abdomen. It should also be considered for pre-menopausal females with a history of recent uterine or invasive cervical procedures. The history of placement of uterine devices (recent or remote) should also raise suspicion for this rare clinical entity in the mind of a surgeon. Similarly, weight loss, abnormal vaginal bleeding, or purulent discharge per vaginum may point towards pelvic pathology. This case report serves to increase the awareness of, as well as the importance of, an entity with which the general or acute surgeon may not be familiar. This report reminds the surgeon to be cognizant of and look for alternate causes/sources when the common causes are absent. Diagnostic laparoscopy makes it easier to visualize even the most inaccessible parts of the abdominal cavity with minimal tissue trauma. With the smaller incisions, it is still possible to lavage the entire peritoneal cavity and place drains in precise locations. Thus, laparoscopy is not only beneficial in determining the origin of acute abdomen but may also allow minimally invasive treatment, decreasing hospitalization, surgical complications, and recovery time. We strongly recommend the use of diagnostic laparoscopy over laparotomy for cases of pneumoperitoneum after other noninvasive tests have been exhausted within the conceptual frame of the clinical scenario.
